# Surface energetics and protein-protein interactions: analysis and mechanistic implications

**DOI:** 10.1038/srep24035

**Published:** 2016-04-06

**Authors:** Claudio Peri, Giulia Morra, Giorgio Colombo

**Affiliations:** 1 Istituto di Chimica del Riconoscimento Molecolare, Consiglio Nazionale delle Ricerche, via Mario Bianco, 9, 20131, Milan, Italy

## Abstract

Understanding protein-protein interactions (PPI) at the molecular level is a fundamental task in the design of new drugs, the prediction of protein function and the clarification of the mechanisms of (dis)regulation of biochemical pathways. In this study, we use a novel computational approach to investigate the energetics of aminoacid networks located on the surface of proteins, isolated and in complex with their respective partners. Interestingly, the analysis of individual proteins identifies patches of surface residues that, when mapped on the structure of their respective complexes, reveal regions of residue-pair couplings that extend across the binding interfaces, forming continuous motifs. An enhanced effect is visible across the proteins of the dataset forming larger quaternary assemblies. The method indicates the presence of energetic signatures in the isolated proteins that are retained in the bound form, which we hypothesize to determine binding orientation upon complex formation. We propose our method, BLUEPRINT, as a complement to different approaches ranging from the ab-initio characterization of PPIs, to protein-protein docking algorithms, for the physico-chemical and functional investigation of protein-protein interactions.

Protein-protein interactions (PPIs) are central to the majority of biological processes, from cell adhesion to immune functions. Hormone-receptor binding, chaperone-regulated client folding, antigen-antibody complex formation and signal transduction (to name a few) are all regulated through a delicate interplay of different physico-chemical factors, ranging from the conformational organization of the interacting surfaces to the evolutionary optimization of their sequences for efficient binding. An improved understanding of PPIs could not only advance our knowledge of chemical biology but also facilitate the rational discovery of new molecules targeting PPIs for therapeutic interventions, as well as design novel protein interactions for research and biotechnological purposes.

Despite the advances of experimental genomic and proteomic efforts producing large amounts of data, combined to the development of databases and bioinformatics algorithms^1^,^2^,^3^,^4^,^5^, there is still a need to investigate and understand at the atomic level the peculiar properties of protein surfaces that underlie protein-protein recognition. Unveiling the identities of protein sites that play a key role in defining the formation of a complex, and the ways in which they are selected for association may assist in inferring their function and dynamic regulation^6^,^7^,^8^,^9^,^10^,^11^,^12^.

In this context, a great effort has been put in computational studies aimed at illuminating the structural or physico-chemical properties that differentiate the regions in the proximity of binding sites. These can be formalized in descriptors that, once implemented in prediction systems, would allow the prediction of interaction interfaces. State-of-the-art PPI prediction methods usually achieve remarkable performance relying on different sets of combined parameters^12^,^13^,^14^,^15^.

In this framework, our group has recently developed a new way to approach *in silico* epitope prediction of protein-antibody and/or peptide-MHC recognition regions based on a novel physico-chemical descriptor of the internal energetics of proteins. At its core, the method employs a matrix of pairwise non bonded interactions, decomposed by Principal Component (PC) analysis and rebuilt according to its principal components (eigenvectors): each element defines the strength of interaction between residue pairs. The matrix is used to identify networks of energetically non-optimized pairwise interactions, meaning subnetworks of aminoacids characterized by a very low non-bonded interaction energy with the rest of the protein. These in turn can define antibody binding sites on an antigen’s three-dimensional structure. Such method is freely accessible as a web-based predictor under the name of BEPPE^16^,^17^,^18^. Another use of the same Energy Decomposition Method (EDM) is the analysis of the energy matrix to highlight hotspots of protein stability^19^.

Here, we hypothesize that the reach of this analysis should not be restricted only to antibody binding sites and T cell receptor recognition. With this in mind, we formulated the hypothesis that the analysis of intra-protein energetic couplings with different intensities can uncover new and more general aspects of protein-protein recognition and interaction.

In this paper, we used and expanded the Energy Decomposition Method to search for those protein surface networks characterized by strong couplings among constituting residue pairs, as well as surface networks characterized by weak couplings among residue pairs. Strong couplings define residue networks important for structural stabilization^19^. Conversely, weak couplings (or low coupling) subnetworks identify regions that are energetically uncoordinated with the rest of the protein and may be more prone to interact with other partners^20^,^21^,^22^,^23^,^24^.

We analyzed 30 binary interactions and their respective 60 constituent monomers, each one available in both crystallographic forms (bound, unbound) in the Protein Data Bank, and verified whether the patches of coupling or uncoupling energies generate specific motifs (marks) across the interfaces. In order to be useful for mechanistic analysis or predictive purposes, the marks need to be persistent and detectable from the constituting proteins, so that the analysis of an isolated molecule can provide information on the bound state.

The results of our analysis revealed that the energetic subnetworks consisting of strongly coupled residues locate preferentially the inner core of the binding sites, a region that is more prone to accommodate PPI hotspots^25^,^26^,^27^, while uncoupled areas tend to gather on the surrounding rim. Thereby, they form distinct patches that closely localize near and across the binding site, although they do not pinpoint a specific substructure defining the interface. Interestinlgy, when considering the structures of the complexes, coupled and uncoupled areas of one partner were observed to co-localize with the respective coupled and uncoupled areas from the other partner, forming continuous motifs crossing the interface of the interacting molecules.

Our approach thus revealed a notable effect of co-localitazion of regions of strong coupling or uncoupling upon complex formation, unexpected *a priori*.

The analysis of such energetic motifs could help identify binding sites and binding sites orientations from the *monomeric, isolated partners* for a diverse ensemble of protein-protein assemblies. As a consequence, we developed an algorithmic implementation of the method for the analysis of monomers and complexes under the name of BLUEPRINT (*BEPPE-Like Un-coupling Energy for PRotein INTeraction*). Examples, applications and impact for further practical and theoretical studies will be discussed.

## Results

### Dataset preparation

The dataset was composed of binary complexes (heterodimers) chosen from the Protein Docking Benchmark 4.0^28^ as a representation of different kinds of proteins and protein-protein interactions. The collection comprises high-resolution structural data of known transient protein-protein interactions, complete with separate PDB codes for the complex and the individual partners. Our dataset features 30 complexes and 60 related monomers (Supplementary Table S1). Among these, four dimers (1F51, 1F6M, 1IB1, 1KKL) are engaged in further quaternary arrangements, providing structural data of three tetramers and one hexamer. In order to portray a consistent representation of the non-bonded energy, we selected complete proteins or integral large domains for the analysis. For the same reason, we excluded those proteins lacking structural information of large sections (gaps) that could not be reasonably reconstructed by molecular modeling (see Methods). The monomers were chosen to represent small to medium-sized proteins (56–470 residues), and we excluded antibody-antigen interactions, considered as a peculiar subcategory of PPIs^29^. The original Benchmark classifies each interaction into three categories: rigid-body, medium and difficult. Rigid-body (123 cases) comprise those proteins that feature minor distortions at the binding site as consequence of binding, so the bound and unbound forms of the two proteins are structurally equivalent for the most part. Medium (29) and difficult (24) cases are defined according to the increasing level of structural distortion of the binding site between bound and unbound state. According to the aforementioned requirements, our dataset was finally composed of 15 rigid-body interactions, 10 medium cases and 5 difficult ones.

### Energetic coupling/uncoupling analysis: BLUEPRINT

**Figure 1:**
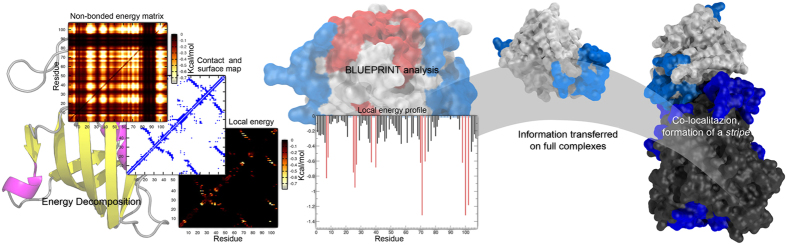
Schematic representation of the workflow beneath the BLUEPRINT analysis. From Left to right, the nonbonded intramolecular and solvation energy terms for each protein have been calculated and retrieved via GBSA, and expressed in form of a pairwise energy matrix via Energy Decomposition analysis. The energy matrix is filtered by a similar, pairwise contact map of surface residues to highlight the local interaction networks. This new matrix is collapsed to a monodimensional profile, averaging all energy contributions for each amino acid. From this energetic profile, the strongest (red peaks) and weakest elements (blue, energy close to 0) are selected to be mapped on the protein surface. Our benchmark is composed of dimers, and the results for each protein (in this case only the weak elements are displayed) are transferred on the full structure of their complex, revealing the effect of co-localization (*blue stripe*). PDB ID: 1B6C

Our protocol describes the energetic coupling as a measure of the local non-bonded energy contribution of surface residues to intraprotein stability. The concept is an expansion of the Energy Decomposition Method (EDM) used for assessing the determinants of stability of protein 3D folds^19^,^30^,^31^ and originates from BEPPE^16^,^17^,^18^, a further derivation of EDM employed in the prediction of immunoreactive epitopes^20^,^21^,^32^. The methodological outline of this study, starting from the atomic coordinates of the proteins and getting to a per-residue representation of the energy distribution in the system, is schematically shown in Fig. 1. We collected the structural information of all proteins in their unbound and bound form (60 + 30 PDB codes), and ran energy minimizations for each of them (see Methods). The energetic determinants were computed as described in Scarabelli *et al.*^16^ using the AMBER 11 software package^33^. The EDM analysis is used at this stage to reconstruct a simplified energy matrix from the non-bonded interaction energy contributions (van der Waals and Coulombic interactions) by the combination of a set of principal eigenvectors selected according to their level of representation of the full energy matrix (see Methods). The resulting matrix (Fig. 1) is filtered by the respective protein contact map, which finally provides the contributions to stability due solely to local interactions.

The final matrix is simplified in the form of a profile, averaging all the energetic contributions for each residue to one value (Fig. 1). From this profile we select those surface residues showing higher energetic coupling, i.e. a strong contribution for the stabilization energy in their local environment (the peaks); and those showing an extremely weak contribution, the uncoupled ones (those residues with energy profile close to 0), according to a cutoff (see Methods and Supplementary Table S4).

After the analysis, all coupled and uncoupled residues are mapped on the 3D structure of monomers and complexes. The primary objective of the analysis is to find patches of surface residues with a shared energetic property in their local networks (coupled or uncoupled) and to map them on the constituent proteins of a complex in search of possible characteristic motifs across the binding interface.

### Continuous marks

The results of the energetic analysis can be mapped on the surface of the isolated proteins. They lead to define patches of strongly interacting residues represented as peaks in Fig. 1 (coupled), and the poorly optimized patches featuring low energetic couplings. Then the energy patterns are transferred from each pair of isolated proteins to the surface of their respective complex, to check for co-localization of patches across the interface between two binding partners.

From now on we will refer to the uncoupled networks as “*blue stripes*”, whenever the motifs co-localize at the interface of the binding partners. Similarly, the strongly coupled patches that form a continuous mark across the interface will be referred as “*red stripes*”, following the color code adopted in the images (Fig. 1). To define colocalization, the minimal distance between the two portions of a stripe (one from each monomer) is 3.8 ± 0.9 Å on average, with a highest distance value of 6.9 Å for complex 1R6Q, *blue stripe*.

Looking at the isolated partners after projecting the results of the analysis onto the structure of the full complex, we assess the presence of a *blue stripe* in the majority of cases (26 out of 30), while the presence of a sharp *red stripe* can be verified in 20 candidates (Table 1). The energy analysis was then repeated on the full structures of the bound complexes, in order to test whether the energetic pattern would be preserved and remain detectable after the binding event. We find *blue stripes* in 20 out of 30 cases and red stripes in 27 out of 30.

Next, we aligned the results of the analysis performed on the monomers over the sequence of their complex and compared the results in terms of sequence identity. We noticed that globally the stripes share an average 66% (blue) and 62% (red) residues between the two cases (Table 1). A control based on random predictions of an identical number of residues selected across each protein surface scored an average 35.65% ± 4.1 and 32.4% ± 4.3 for uncoupling and coupling analysis respectively (1000 random replicas). It is worth noting that the number of residues selected by BLUEPRINT for each pair of proteins in the two analyses, e.g. in isolation versus in the context of the complex, remains comparable, supporting the idea that these networks are persistent upon binding or in isolation, although with differences.

There are only six cases for which a stripe cannot be assessed in either condition (monomers and complexes), and they comprise 4 dimers lacking a *blue stripe* and 2 lacking a red one. If we consider the absence of a stripe in only one of the two conditions, the only candidates for which is impossible to assess both *blue* and *red stripes* are 1KTZ and 1FFW. Overall, there is a prevalence of dimeric complexes featuring a full, concomitant *blue* and *red* coordination (11 cases). Dimers featuring just one of the two coupling effects at the interface are also common, 10 for the *blue stripes* and 7 for red ones.

Interestingly, the results lacking a *stripe* are evenly distributed among the three categories in which the dataset is subdivided (rigid-body, medium, difficult, see Dataset preparation), and since the entries in each category are sorted by number of residues (details in Supplementary Table S1), the absence of continuity in the marks is not apparently associated with conformational changes or the increase in protein size.

### Visual inspection

The results of the BLUEPRINT analysis reported above indicate the presence in the isolated proteins of patches of surface residues sharing the same energetic property in their local coupling networks that, when considering the respective complexes, form a continuous mark spanning the constituent partners.

**Figure 2:**
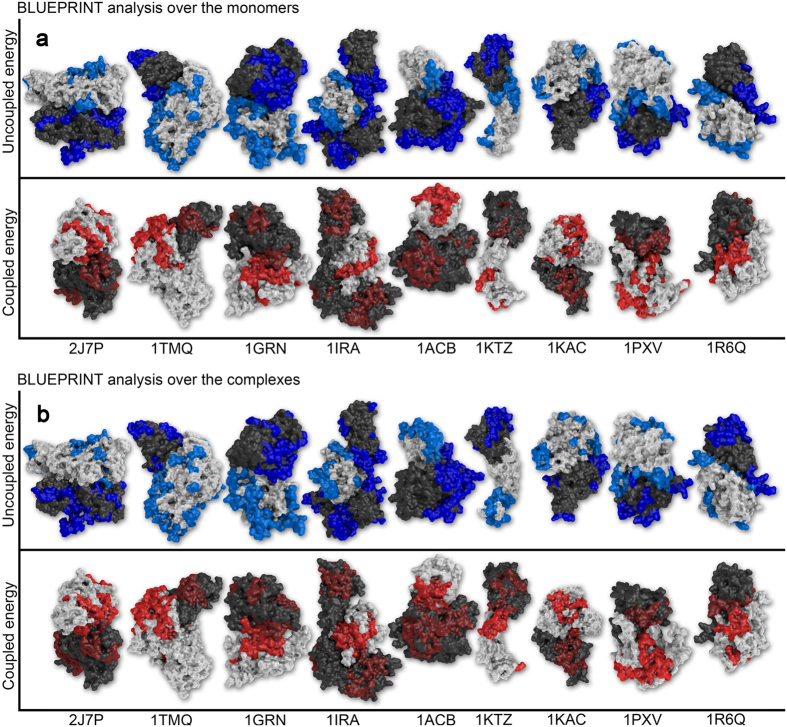
Visual representation of BLUEPRINT results from the analysis of 18 monomers and 9 complexes, mapped on the 3D structure of the full dimers. The upper (**a**) and lower (**b**) panels show the same 9 complexes arranged in the same order according to the PDB ID located on the bottom line. In order to display the results clearly, we rotated the complexes (90 or 180 degrees), so upper and lower complexes appear different. The two proteins composing each dimer are identified by a different color (white and dark gray). On top we show the regions characterized by energetic uncoupling in their local network. Light blue regions belong the white monomer, while deep blue regions belong to the dark one. The lower panel shows the surface patches characterized by strong energetic coupling. The color code adopted here is similar: results mapped on the white protein are highlighted in bright red, whereas a darker shade has been used for the grey protein. All molecules are rendered with The PyMOL Molecular Graphics System, Version 1.7.4 Schrödinger, LLC.

A graphical representation of the results is reported in Fig. 2 as a subset of 18 proteins (top panel, analysis over the monomers) and 9 complexes (bottom one, analysis over the complexes). The complete set of images for the remaining proteins and complexes is reported in Supplementary Figures S1 and S2).

Visual inspection of the monomers highlights three main elements: first, the uncoupled areas actually define “*stripes*” on the protein surfaces, putting together residues that are not necessarily adjacent on the primary sequence. A *stripe* is observed among local low energy coupling areas (*blue stripes*) in the majority of cases (26/30 monomers and 20/30 complexes), producing an interesting geometrical continuity (e.g. 2J7P, 1ACB, 1R6Q). The red *stripes*, on the other hand, are sparser though present in the majority of cases (20/30 monomers, 27/30 complexes).

The second element is the size of the *stripes*. The *blue stripes* are not limited to the interface region, but they tend to expand across the protein surface (e.g 1GRN, 1IRA, 1ACB). Conversely, red *stripes*, indicating spots of high energy coupling, are smaller, and usually confined across the interface area.

The third characteristic indicates that *blue stripes* and *red stripes* tend to gather on opposite regions of the protein/complexes (e.g. 1TMQ, 1KAC, 1R6Q).

The comparison with the BLUEPRINT analysis run on the entire complexes highlights additional interesting elements. The first difference regards signal definition. The analysis performed on the monomers returned well-defined *blue stripes* and sparser red ones. In the complex analysis we have the opposite situation. The *blue stripes* are still visible for the most part, but they are less refined. Red stripes conversely are clearer, less sparse and characterized by higher continuity. This is consistent with the previous observation concerning the number of cases showing continuity, with uncoupled regions forming more *blue stripes* when monomers are analyzed compared with the same analysis over the complexes, vice versa for red *stripes* (Table 1). Overall, the effect of energetic uncoupling appears to be more defined when the protein is in individual state, while the strong energetic couplings emerge when calculated on the bound state.

The areas selected by BLUEPRINT in the two conditions are very similar, but we appreciate a loss in the number of uncoupled residues upon binding, especially at the interface (e.g. 2J7P, 1GRN, 1KTZ, 1R6Q). These residues may switch to highly coupled behavior instead (e.g. 1ACB, 1KTZ). The effect is interesting but not unexpected: The BLUEPRINT analysis is dependent on the local network one residue establishes with the amino acids in its proximity. In the event of protein binding, the network of one amino acid can change dramatically in the proximity of the binding interface, and as a consequence of conformational rearrangements.

Importantly, focussing on conformational rearrangements, it is very interesting to notice the case of 1IRA, composed of proteins 1G0Y and 1ILR (Fig. 2). In the Figure, 1G0Y is depicted as dark and is subdivided in two lobes. 1ILR is represented as white, and rests encapsulated between the lobes of its partner. 1GOY is actually different in its monomeric structure because the lobes are closed and in mutual contact. Even if affected by such a conformational change, the results on the monomer (thus the closed structure) recapitulate the results returned on the full, open complex, with an identity of 78.2% for uncoupled areas and 75.9% for coupled ones, enough to produce a *blue stripe*. Importantly, other proteins for which a major conformational rearrangement is present (all *difficult* cases, 2O3B, 1F6M, 1PXV, 2C0L) also show stripe continuity (Table 1, Fig. 2, Supplementary Figures S1, S2).

### Tetramers and Hexamers

As previously stated in the dataset preparation section, the analysis was focussed on binary interactions involving heterodimers, but included four cases presenting a further supramolecular organization in which the original dimer is engaged with identical copies in symmetrical assemblies (three tetramers and one hexamer). We applied the same methodology described for the dimers to those proteins of the dataset for which a higher supramolecular organization level was present, since proteins engaged in transient interactions are commonly promiscuous or part of larger protein complexes. These cases (1F51, 1F6M, 1IB1, 1KKL) represent a first valuable opportunity to test the hypothesis of the conservation of the properties of patches and stripes from the monomeric state to a higher level of complexity.

**Figure 3:**
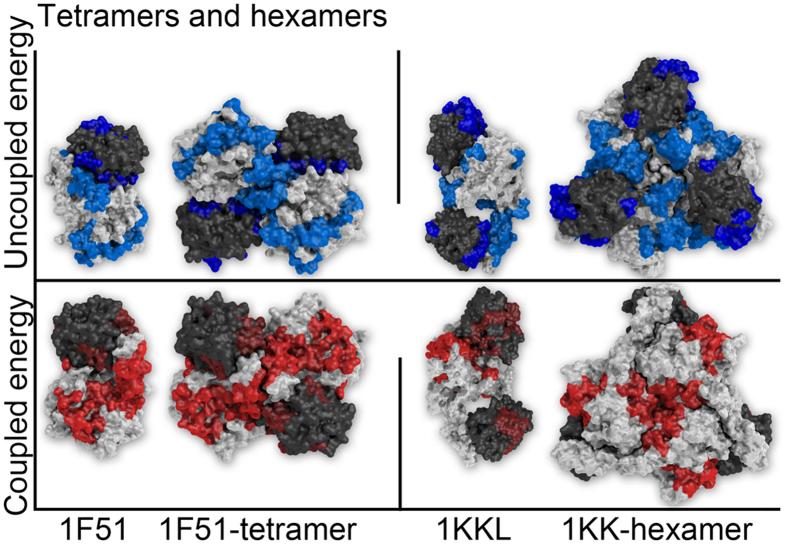
Visual representation of BLUEPRINT results mapped on the 3D structure of their supramolecular complexes. In this figure we show the result of BLUEPRINT analysis run over the individual proteins composing complex 1F51 (1IXM, 1SRR, left panel) and 1KKL (1JB1, 1HPR, right panel). The two proteins composing each complex are identified by a different color (white and dark gray). The uncoupled (blue) and coupled (red) energy patches are mapped on the structure of their partial complex (dimer/trimer) and their tetrameric/hexameric form. In order to display the results clearly, all “coupled” views are rotated 180° with respect of their corresponding “uncoupled” ones.

In this framework, we mapped the analysis of the two monomers composing each complex over the 3D structure of the respective quaternary 3D structures. Overall, all four cases returned a *blue stripe*, and three out of four complexes featured a *red stripe*, with the exception of 1IB1 (Supplementary Table S2). In Fig. 3, we show the result of this operation using complexes 1F51 and 1KKL.

As reported in figure, the coupled and uncoupled patches calculated starting from the structure of single proteins form very sharp *stripes* when placed together on the full assembly, with continuity across all chains. *Blue stripes*, especially, are located in the close proximity of the binding interfaces, while the red ones tend to form patterns across the outer surface. In Fig. 3 we added a second structure for comparison, the monomer data mapped on the partial complex (dimer, or trimer in the case of 1KKL). The comparison shows another interesting element: the continuity of the *stripes* is not immediately clear when looking at the partial structures, and the full patterns emerge only in the full complex.

Finally, following the same protocol we also mapped the dimer analysis (complex) over the full supramolecular assemblies, returning a full 4/4 *blue stripes* and red ones (Supplementary Table S2) and confirming the previous observations for visual similarity between the two analyses.

### Stripe Composition

**Figure 4:**
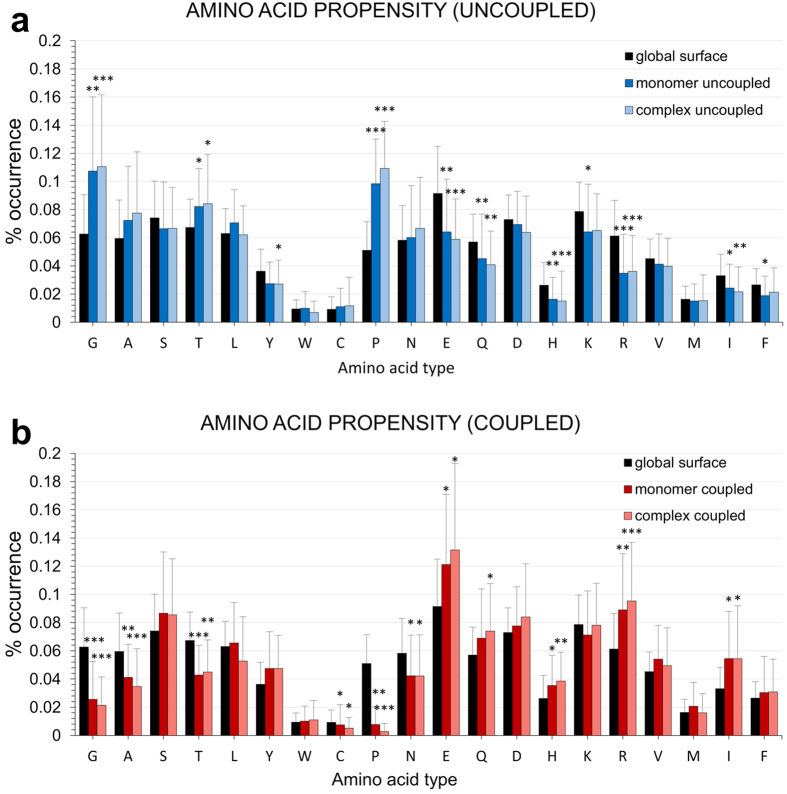
Overall occurrence propensity of each amino acid into BLUEPRINT results. The percentage of occurrence of each amino acid as part of an **(a)** uncoupled patch (blue, upper panel) or **(b)** a coupled one (red, lower panel) is displayed compared with the occurrence of each amino acid considering the whole surfaces. Each bar represents the average occurrence across the dataset with standard deviation shown as error bars. Each panel distinguish between the results of monomer analysis (deep blue/red) and analysis over the complexes (light blue/red). Significance of monomer and complex results distribution against the surface were evaluated with two tailed Mann-Whitney U test; *p < 0.05, **p < 0.01, and ***p < 0.001 values. Blank, not significant.

The BLUEPRINT analysis focuses on the local, non-bonded coupling energy connecting the surface residues of a protein. To further characterize the physico-chemical properties of the identified *stripes*, we evaluated the identities of their constituting aminoacids. We thus compared the residues selected by BLUEPRINT with the overall aminoacid composition of the protein surfaces, calculating the percentage of occurrence of each amino acid as part of an uncoupled patch (*blue stripe*) or a strongly coupled one (*red stripe*) with respect to the occurrence of the amino acid in the whole surfaces. Figure 4, represents with bars the average occurrence of each amino acid across the dataset with standard deviation shown as error bars.

The returned propensities are very similar comparing monomer and complex analyses (Fig. 4, Supplementary Table S3). The distributions present large deviations, consistent with the heterogeneity of the dataset that accounts for different types of proteins and interactions. Interestingly, some residues show a dramatic change in their occurrence compared to the surface, and even more between coupled and uncoupled residues. The presence of Glycines and Prolines is favoured in uncoupled patches, vice versa it is unfavorable in coupled ones. Glycine lacks a full side chain, and Proline is a rigid aminoacid presenting a limited surface to interactions, making the two amino acids favoured in uncoupled patches. Consistent with this observation, the comparison also shows a trend to enrich uncoupled patches with small residues (Alanine and Threonine). In contrast, large hydrophobic together with large and highly charged aminoacids (such as Glutamate, Arginine and Isoleucine) are overrepresented in strongly coupled patches. Interestingly, the latter are often found to define the contacting regions of interacting proteins^34^, as well as the PPI hotspots giving the highest contribution to the stability of the interaction surfaces.

## Discussion

Understanding the processes underlying protein-protein interactions (PPIs) at the molecular level is important for both fundamental and practical issues. From the fundamental point of view, it can help understand the correlations between sequence, structure and function as well as reveal the molecular basis for the (dis)regulation of important biochemical pathways. From the practical standpoint, it could set the basis for the design of bioactive molecules, to develop biosensors or to engineer probes for the discovery and validation of disease targets. Interesting examples of such applications are the realization of protein mimics of antigenic epitopes^35^, or the design of reagents for protein targeting^32^.

Systematic studies of transient interactions have highlighted key factors for the formation and stabilization of protein complexes, including geometric complementarity, hydrophobicity, solvent accessibility or desolvation propensity^36^,^37^.

In this paper, we approached another physico-chemical aspect of interacting proteins, studying the interplay between the interaction networks of surface residues in isolated proteins and their roles in respective protein complexes. The initial hypothesis entailed the presence of specific intramolecular energetic signatures for the residues participating in the interaction process. This is based on the results of a recently developed computational approach that proved successful in the prediction of antibody binding sites^20^,^21^,^22^,^23^,^24^. We focussed our analysis on local networks of strongly coupled residues, carrying a relevant contribution to the conformational stability of the protein region they belong to, and local networks of minimally coupled amino acids, representing residues with the lowest contribution to the intraprotein stabilization energy. When analyzing single proteins in isolation, these networks turned out not to correspond exactly to the interface observed in the X-ray structures of the complexes (which is defined as such only upon complex formation), but to identify continuous patches connecting the binding partners when projected on the structure of their respective complexes. We called these patches “*stripes”* (*red* and *blue stripes)*, and carried out further investigations. Indeed, from the analysis of a heterogeneous dataset of known interacting proteins, we assessed the co-localization of the patches identified in the individual proteins once they are displayed on their respective complexes as a general property. The whole protocol has been named BLUEPRINT (*BEPPE-Like (Un)coupling Energy for PRotein INTeraction*).

Energetically non-optimized areas (corresponding to local networks of minimally coupled amino acids) defining the rims of the binding surfaces, are conserved on passing from the isolated proteins to their complexes, where they end up forming continuous stripes spanning the two contacting macromolecules.

In addition to the observations made possible with a dataset of dimeric interactions, the general principle of our model is supported by results obtained for proteins forming larger tetrameric and hexameric multimers: the large number of patches observed for these proteins in isolation indicates the presence of several possible binding sites for the formation of polymeric supramolecular aggregates (Fig. 3), extending the continuity of the stripes beyond the simple binary level. This fascinating possibility needs to be carefully considered in future tests.

Another aspect worth of attention is the apparent independency of the method from the effects of large conformational re-arrangements. Using the original classification of protein Benchmark 4.0, rigid-body cases are desired since they limit the influence of conformational perturbations during the initial validation stage, while medium and difficult cases are useful to test the robustness of the analysis in the presence conformational displacement of the interface. According to our results, the ability of the method to highlight co-localized *stripes* with high identity between monomers and complex is unchanged in the latter categories (Table 1). This reinforces the concept of a constitutive energetic effect, which is not simply lost while the protein is isolated or undergoing structural rearrangements.

**Figure 5:**
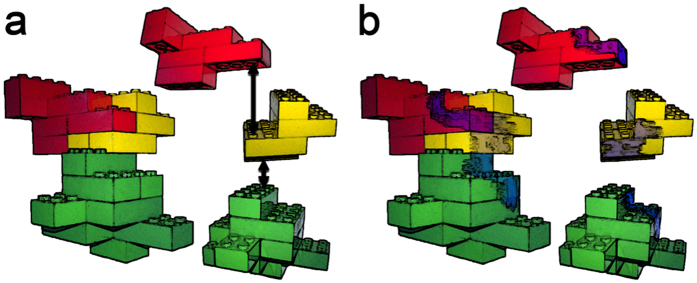
Conceptual representation of our theoretical strategy, expressed by toy bricks analogy. (**a**) The traditional approach to protein-protein interaction pursues the location of the binding sites and use them as guidelines for assembly. In our strategy (**b**) a mark on the complex persistent on each individual part would be able to reveal additional information, such as the binding orientation.

The vast majority of computational methods devoted to the analysis and the prediction of PPIs focuses on the identification of the binding interface. The reason for this is the benefit of a direct, non-ambiguous source of information that can be annotated and retrieved. This can be compared to an “instruction manual”: to know exactly where two proteins assemble is the best starting point for the reconstruction of the complex *a priori* (Fig. 5A). Yet, it may not be the only one. Using a familiar and simple analogy, one can imagine somebody (e.g. a kid) leaving a mark of watercolor on an assembly of toy bricks. If the mark is persistent, it will remain visible on its constituent parts even after disassembly. Based on this mark, one may be able to reconstruct the original assembly following the continuity and the orientation of the color, perhaps even in absence of instructions (Fig. 5B).

**Figure 6:**
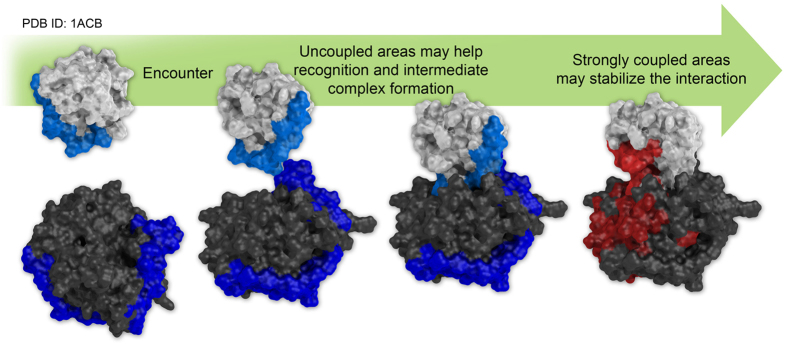
A proposed hypothetical model connecting surface energetics and protein binding. According to our data, networks of interacting residues bearing a collective strong or weak energetic contributions (coupled and uncoupled respectively) may play a role during the binding event. From left to right, during the encounter process the uncoupled areas (blue) may facilitate the formation of the intermediate complex, thus leaving an enhanced uncoupled signature on the surface of isolated proteins that can be revealed in the form of a *blue stripe* by BLUEPRINT analysis. Conversely, strongly coupled areas (red) may help the formation of a stable interaction, so their coordinated signal, once again visibile by BLUEPRINT analysis in the form of a *red stripe*, becomes prominent when calculated over the structure of already formed complexes.

A similar perspective may be adopted for the PPI problem: according to our observations, protein adaptation to their binding partners may have left such a trace in the energetics of the surfaces of the partners during the course of evolution, determining networks of energy-couplings of different intensities around and across the final binding interfaces. In this context and in the conceptual framework of the current two-steps (dock-and-lock model) kinetic model^36^,^37^,^38^,^39^,^40^ for protein-protein recognition, energetically unoptimised regions may drive the initial formation of the complex (dock), and the high energy contacts may then play a role in the stabilization of the bound state (lock). In the monomeric state, networks of amino acids that are minimally coupled intramolecularly with the rest of the protein may indeed support conformational changes, be recognized by and adapt to a binding partner with minimal energetic expense, favouring the initial encounter of the two interacting partners. Such initial complexes, may visit a (certain) number of conformations that eventually evolve^41^,^42^ to the final, experimentally observed, complex structure. In this line of thought, we speculate that non-optimized networks represent the soft-spots where the proteins first come into contact, aiding the formation of a starting binding complex (Fig. 6). Once formed, the complex could aptly evolve towards the final conformation observed in experimentally determined structures. The redistribution of interactions in the latter structures, specifically those located near the binding site, modifies the networks, determining in most cases stronger energetic couplings, which contribute to stabilize the complex (Fig. 6).

This hypothesis is corroborated by the differences observed between blue and red stripes in monomer vs. complex analysis. Blue stripes are clearer when calculated on isolated structures, while red stripes are well-defined in the actual protein-protein complexes, and include regions across the binding interface that were previously uncoupled in the monomeric form.

We propose that the persistent motifs, isolated in the energetic footprint of the constituent proteins in their monomeric isolated form, define a signature property of proteins involved in protein-protein interactions. To further explore this hypothesis, in a possible future evolution of this work, one could analyze the presence of such patterns on proteins not involved in PPIs (such as enzymes binding small organic molecules or metabolites), as well as look for similarity in the stripe morphology of structurally homologue proteins, possibly correlating it to functional similarity.

In conclusion, the proposed method provides interesting novel insights into the salient aspects of protein energetics that determine protein-protein interactions. The central concept of the approach we presented entails a shift from interface-focused prediction to an energy based pattern analysis. This could represent a complementary method to classical interface predictors and docking algorithms to gain information on the binding process, including the location of binding sites, the properties of their surroundings and how these can preferentially orient the binding partners. Energetic coordination carries some interesting theoretical implications too, posing new questions, such as the investigation of the link between non-bonded energy networks and co-evolution^11^,^43^. Finally, we think that the concept in its simple yet innovative premise holds wide promises for improvement and refinement.

## Methods

### Molecular modeling

Each protein (and complex) was prepared for analysis by conversion of selenomethionines (MSE) to methionine and modeling of missing sidechains using MAESTRO version 9 from SCHRÖDINGER software and the package Prime^44^,^45^,^46^. Missing loops were modeled according to the same instruments, with except for proteins lacking significant portions that cannot be realistically computed in absence of a reference template. In those cases, the gap was filled by homology modeling employing templates ranging from high to total identity to the query (usually alternative structures of the same proteins) using the molecular modeling environment MAESTRO.

### Minimization and GBSA decomposition

All input structures were converted in AMBER-compatible PDB files (Assisted Model Building with Energy Refinements)^33^, and Molecular Mechanics (MM) parameters were generated according to the force field ff99SB^47^. The structures were undergone a preliminary energy minimization procedure, consisting of 500 minimization steps with the steepest descent optimization algorithm. The resulting models were treated in implicit solvent by means of the MM-GBSA calculation approach (*Molecular Mechanics Generalized Born Surface Area*) included in the AMBER 11 software package. The polar solvation term was approximated with the Generalized Born (GB) model^48^,^49^ and OBC rescaling^50^ for solvation energy. The dielectric constant was set to 80 for the bulk water and 1 for the protein and internal cavities and we adopted a physiological salt concentration (0.1 M). The nonpolar solvation term was calculated through the evaluation of the solvent accessible surface area (SA) using the icosahedra approximation. The nonpolar contribution to the free energy of the free energy of solvation was calculated as Enp = surften*SA (no offset correction) and the surface tension used is 0.0072 cal/mol/Å^2^.

### BLUEPRINT analysis

The electrostatic and Van der Waals interaction terms from GBSA decomposition were used to build a matrix composed of the interaction energy between residue pairs in the structure. The matrix was decomposed through Principal Component Analysis. The resulting main eigenvectors and eigenvalues were used to recover a simplified matrix recapitulating the most relevant interactions within the protein structure.

The first eigenvector recapitulates the main contribution to the stabilization energy, which is due to the strongest pair interactions defining the energy core. In the case of small, monodomain proteins, it is typically sufficient to correctly identify the residues constituting the core. In contrast, in the presence of more complex topologies or multi domain proteins, more eigenvectors in the energy approximation can effectively improve the definition of the energy core. The relevant eigenvectors are those containing multiple consecutive components of equal sign, which points to the presence of a cluster of similarly interacting residues. To select them, for each eigenvector 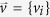 the lag-l autocorrelation of the component series {*v*_*i*_} is calculated:


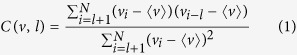


and all eigenvectors with a non zero lag-l autocorrelation for l lower than a threshold value are selected. The threshold, adjusted to yield optimal energy coverage among the broadly heterogeneous dataset, was set to 30.

The new and simplified matrix was filtered by a contact map, in order to remove all distal contributions. The topology is defined reducing each residue to a pair of coordinates: the Cα carbon (CA) and the center of mass of the sidechain (CM). Each pair of residues (*R*_*i*_*, R*_*j*_) is assigned a positive value (1) in the contact map if any of these two elements are in proximity (CA_*i*_-CA_*j*_, CA_*i*_-CM_*j*_, CM_*i*_-CA_*j*_, CM_*i*_-CM_*j*_) within a radius of 6 Å, otherwise the contact is flagged as negative (0). At the same time, the contact map was used to restrict the analysis to the surface residues only. The solvent accessibility for each residue (SASA) was calculated with the software naccess^51^, and a negative flag has been assigned to any residue with SASA <12 Å^2^.

The newly reconstructed local energy matrix of surface residues is then collapsed into a linear per-residue profile, averaging all local contributions for each amino acid. The residues were subsequently ranked according to the resulting energy contribution. The selection of residues carrying the weakest or strongest coupling energy is defined by means of a cutoff. We varied the cutoff by considering the interval between 10 to 40% of surface residues at increasing intervals of 5% and selecting the threshold first displaying clearly the continuous effect within this range in both monomers and complex analysis. The analysis produced well-defined and continuous patches preferentially in the range of 30–40% for uncoupled surface residues, while strongly coupled areas display stripes in the range of 25–35% (Supplementary Table S4). Averaging the threshold distribution obtained for our protein dataset, we consider the top 30% stabilizing residues as a consensus cutoff to define the strongly coupled set in the vast majority of cases, and 35% as the consensus cutoff for minimally coupled amino acids.

In order to compare the monomer results with complex analysis, BLUEPRINT maps the monomer patches on the sequence and 3D structure of the complex using the Needleman-Wunsch algorithm for a global optimal alignment^52^ (to account for occasional sequence dissimilarities or different terminal ends).

## Additional Information

**How to cite this article**: Peri, C. *et al.* Surface energetics and protein-protein interactions: analysis and mechanistic implications. *Sci. Rep.* **6**, 24035; doi: 10.1038/srep24035 (2016).

## Supplementary Material

Supplementary Information

## Figures and Tables

**Table 1:**
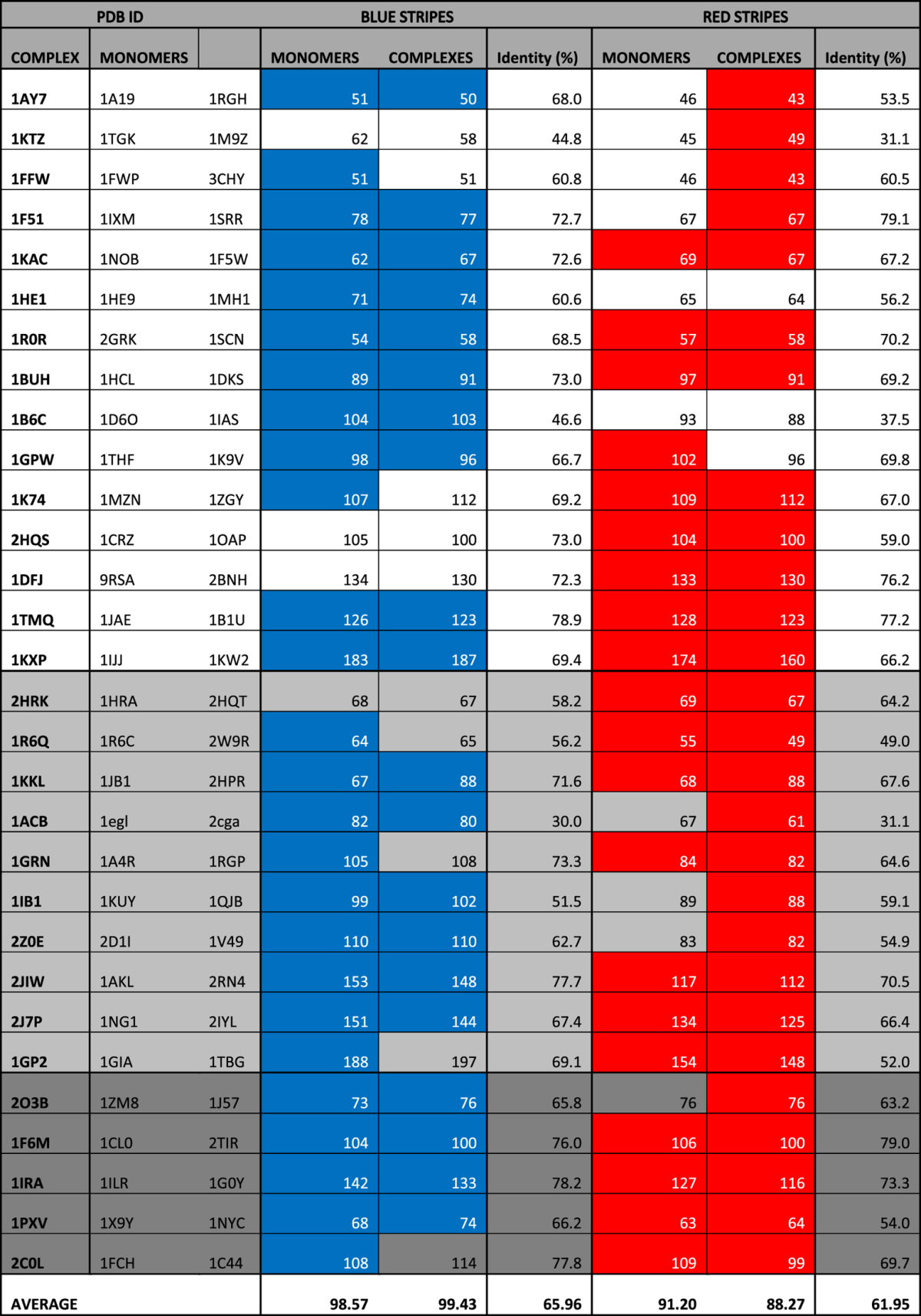
Summary of BLUEPRINT analysis over 60 monomers and 30 complexes.

From left to right, the table reports the PDB ID of each complex and their constituting monomers. The next two columns report the result of energetic uncoupling calculated on the monomers (first column) or the full complexes (second one). A blue background indicate a continuous mark across the binding partners (*blue stripe*). Each cell displays the number of residues selected as uncoupled. The next column (IDENTITY %), indicate the percentage of same-residues between monomers and complexes among the selected amino acids. Similarly, the last three columns show the highly coupled results among monomers and complexes, their *red stripes* and the identity ratio. From Top to bottom: the dataset is subdivided in three categories as listed in the Benchmark 4.0, represented with three shades of gray (white: rigid-body; light grey: medium; dark grey: difficult). Each category sorts the complexes according to their combined number of residues, thus having the smaller complexes on top and the larger ones at the bottom. Refer to Supplemenntary Table S1 for details on the dataset.
