# Supplementary material for: Surface energetics and protein-protein interactions: analysis and mechanistic implications
Source: Sci Rep. 2016 Apr 6;6:24035. doi: 10.1038/srep24035 (PMC4822145; doi:10.1038/srep24035)
Supplement: Supplementary Information [file srep24035-s1.pdf]

Surface energetics and protein-protein interactions: analysis and mechanistic implications

Claudio Peri, Giulia Morra, Giorgio Colombo.

SUPPLEMENTARY INFORMATION

PDB ID	Monomer 1			Monomer 2				
**COMPLEX**	**PDBid 1**	**Length**	**Description 1**	**PDBid 2**	**Length**	**Description 2**	**Type**	**Difficulty**
**1AY7**	1A19	89	Barstar	1RGH	96	RNase Sa	E	Rigid-body
**1KTZ**	1TGK	112	TGF-beta	1M9Z	111	TGF-beta receptor	O	Rigid-body
**1FFW**	1FWP	139	Chemotaxis protein CheA	3CHY	128	Chemotaxis protein CheY	O	Rigid-body
**1F51**	1IXM	179	Sporul. resp. factor B	1SRR	120	Sporul. resp. factor F	O	Rigid-body
**1KAC**	1NOB	185	Adenovirus fiber knob protein	1F5W	124	Adenovirus receptor	O	Rigid-body
**1HE1**	1HE9	134	Pseudomonas toxin GAP dom.	1MH1	186	Rac GTPase	O	Rigid-body
**1R0R**	2GRK	56	Subtilisin carlsberg	1SCN	274	OMTKY	E	Rigid-body
**1BUH**	1HCL	298	CDK2 kinase	1DKS	76	Ckshs1	O	Rigid-body
**1B6C**	1D6O	107	FKBP binding protein	1IAS	330	TGFbeta receptor	O	Rigid-body
**1GPW**	1THF	253	HISF protein	1K9V	200	Amidotransferase HISH	O	Rigid-body
**1K74**	1MZN	240	RXR-a	1ZGY	272	PPAR-gamma	O	Rigid-body
**2HQS**	1CRZ	403	TolB	1OAP	109	Pal	O	Rigid-body
**1DFJ**	9RSA	124	Ribonuclease A	2BNH	456	Rnase inhibitor	E	Rigid-body
**1TMQ**	1JAE	470	alpha-amylase	1B1U	122	RAGI inhibitor	E	Rigid-body
**1KXP**	1IJJ	371	Actin	1KW2	453	Vitamin D binding protein	O	Rigid-body
**2HRK**	1HRA	80	Glutamyl-t-RNA synthetase	2HQT	124	GU-4 nucleic binding prot.	O	Medium
**1R6Q**	1R6C	143	Clp protease subunit ClpA	2W9R	108	Clp adaptor protein ClpS	O	Medium
**1KKL**	1JB1	205	Hpr kinase C-ter domain	2HPR	87	Hpr	E	Medium
**1ACB**	1egl	70	Eglin C	2cga	245	Chymotrypsin	E	Medium
**1GRN**	1A4R	191	CDC42 GTPase	1RGP	242	CDC42 GAP	O	Medium
**1IB1**	1KUY	207	Serotonin N-acetase	1QJB	245	14-3-3 protein	O	Medium
**2Z0E**	2D1I	398	Cysteine protease Atg4B	1V49	120	Microtubule-assoc. 1A/1B	O	Medium
**2JIW**	1AKL	470	Alkaline metalloprotease	2RN4	106	Proteinase inhibitor	E	Medium
**2J7P**	1NG1	294	SRP GTPase Ffh	2IYL	284	Cell division protein FtsY	O	Medium
**1GP2**	1GIA	353	Gi-alpha	1TBG	408	Gi-beta, gamma	O	Medium
**2O3B**	1ZM8	259	NucA nuclease	1J57	143	NuiA nuclease inhibitor	E	Difficult
**1F6M**	1CL0	320	Thioredoxin reductase	2TIR	108	Thioredoxin 1	E	Difficult
**1IRA**	1ILR	145	IL-1 receptor antagonist protein	1G0Y	310	IL-1 receptor	O	Difficult
**1PXV**	1X9Y	357	Cystein protease	1NYC	111	Cystein protease inhibitor	E	Difficult
**2C0L**	1FCH	368	PTS1 and TRP region of PEX5	1C44	123	SCP2	O	Difficult

Table S1. **Dataset of interacting proteins for energetic coupling/uncoupling analysis**: The list includes 30 complexes and 60 monomers subdivided in three categories by conformational difference between bound and unbound form: rigid-body, medium and difficult, as described in the original Benchmark 4.0 (shades of grey and last column). Each category sorts the entries by overall size of the complex (in terms of residue numbers), and for every complex the PDB ID is listed along with the PDB accession of the constituent proteins in their monomeric form, their amino acidic length, and a brief description. The "Type" column qualify the interaction as an Enzyme/inhibitor one (E) or other interfaces (O).

COMPLEX	MONOMERS		MONOMERS	COMPLEXES	ORGANIZ.	MONOMERS	COMPLEXES
**1F51**	1IXM	1SRR			TETRAMER		
**1KKL**	1JB1	2HPR			HEXAMER		
**1IB1**	1KUY	1QJB			TETRAMER		
**1F6M**	1CL0	2TIR			TETRAMER		

Table S2: **Addendum to Table 1, recapitulating the presence of stripe continuity in those proteins undergoing further supramolecular organizations**. A blue or red cell indicate the presence of a discernible blue or red stripe along the tetramer/hexamer, starting from the data collected by the analysis if single monomers or dimers (complexes). Unlike Table 1, The rate of identity between monomer and complex analyses is not reported. Being this case a mapping of the results onto multiple copies of the same proteins, the ratio would be exactly the same.

	SURFACE		MONOMER BLUE			COMPLEX BLUE			MONOMER RED			COMPLEX RED		
	MEAN	SD	MEAN	SD	U-test p-value	MEAN	SD	U-test p-value	MEAN	SD	U-test p-value	MEAN	SD	U-test p-value
**G**	0.063	0.028	0.107	0.053	**1.028E-03**	0.111	0.051	**8.652E-05**	0.026	0.027	**2.921E-06**	0.021	0.020	**6.328E-08**
**A**	0.060	0.027	0.072	0.038	**2.140E-01**	0.078	0.044	**1.086E-01**	0.041	0.024	**9.233E-03**	0.035	0.027	**7.063E-04**
**S**	0.074	0.026	0.066	0.033	**2.836E-01**	0.067	0.029	**2.487E-01**	0.087	0.043	**4.551E-01**	0.086	0.040	**3.040E-01**
**T**	0.067	0.020	0.082	0.027	**1.528E-02**	0.084	0.035	**4.509E-02**	0.043	0.021	**5.227E-05**	0.045	0.023	**2.826E-04**
**L**	0.063	0.018	0.071	0.024	**1.783E-01**	0.062	0.021	**9.882E-01**	0.066	0.029	**7.337E-01**	0.053	0.031	**1.690E-01**
**Y**	0.036	0.016	0.027	0.015	**6.447E-02**	0.027	0.017	**2.854E-02**	0.048	0.026	**6.134E-02**	0.047	0.023	**5.089E-02**
**W**	0.009	0.006	0.010	0.012	**5.394E-01**	0.007	0.008	**1.077E-01**	0.010	0.011	**8.810E-01**	0.011	0.014	**8.166E-01**
**C**	0.009	0.009	0.011	0.013	**8.088E-01**	0.012	0.020	**2.797E-01**	0.007	0.014	**4.825E-02**	0.005	0.008	**2.194E-02**
**P**	0.051	0.020	0.098	0.032	**1.020E-07**	0.109	0.033	**6.783E-09**	0.008	0.012	**2.645E-10**	0.003	0.006	**6.119E-12**
**N**	0.058	0.025	0.060	0.037	**8.941E-01**	0.067	0.036	**3.364E-01**	0.042	0.029	**2.861E-02**	0.042	0.029	**1.727E-02**
**E**	0.091	0.034	0.064	0.038	**2.887E-03**	0.059	0.029	**3.169E-04**	0.121	0.050	**2.234E-02**	0.132	0.061	**1.008E-02**
**Q**	0.057	0.020	0.045	0.032	**9.441E-03**	0.041	0.024	**3.567E-03**	0.069	0.035	**2.337E-01**	0.074	0.034	**3.083E-02**
**D**	0.073	0.017	0.069	0.024	**4.594E-01**	0.064	0.026	**1.202E-01**	0.078	0.028	**2.902E-01**	0.084	0.038	**3.789E-01**
**H**	0.026	0.016	0.016	0.016	**5.626E-03**	0.015	0.021	**7.846E-04**	0.035	0.021	**2.453E-02**	0.039	0.020	**7.401E-03**
**K**	0.079	0.021	0.064	0.034	**4.588E-02**	0.065	0.026	**5.182E-02**	0.071	0.031	**4.202E-01**	0.078	0.030	**9.352E-01**
**R**	0.061	0.025	0.035	0.028	**5.526E-04**	0.036	0.026	**4.564E-04**	0.089	0.040	**3.842E-03**	0.095	0.042	**6.705E-04**
**V**	0.045	0.014	0.041	0.021	**3.590E-01**	0.040	0.020	**1.332E-01**	0.054	0.024	**1.135E-01**	0.049	0.027	**4.160E-01**
**M**	0.016	0.009	0.015	0.012	**5.334E-01**	0.015	0.018	**1.882E-01**	0.021	0.017	**4.362E-01**	0.016	0.014	**6.831E-01**
**I**	0.033	0.015	0.024	0.017	**4.340E-02**	0.022	0.018	**4.271E-03**	0.055	0.033	**1.408E-02**	0.054	0.038	**2.458E-02**
**F**	0.026	0.012	0.019	0.014	**1.752E-02**	0.021	0.017	**1.470E-01**	0.030	0.026	**7.561E-01**	0.031	0.023	**9.941E-01**

Table S3: **significance for Mann-Whitney test comparing the distribution of occurrence of each residue in different *stripes* compared to the overall surface**. The U-test has been applied to the occurrence of each amino acid in each BLUEPRINT testing condition (from left to right: *blue stripes*, monomers; *blue stripes*, complexes; *red stripes*, monomers; *red stripes*, complexes) compared to the distribution of occurrence across all 30 complex surfaces (n=30). The U-test was chosen as a nonparametric test to compare independent sets of occurrence data, which not necessarily follow a normal distribution and is suitable for a small data set. The null hypothesis consider the two distributions (occurrence on the surface / on the *stripe*) as part of the same population. For each testing condition, the averages of occurrence and standard deviations are reported

PDB ID			CUTOFF FOR UNCOUPLED		CUTOFF FOR COUPLED	
**COMPLEX**	**MONOMERS**		**MONOMERS**	**COMPLEXES**	**MONOMERS**	**COMPLEXES**
**1AY7**	1A19	1RGH	35%	35%	30%	30%
**1KTZ**	1TGK	1M9Z	35%	35%	30%	30%
**1FFW**	1FWP	3CHY	35%	35%	30%	30%
**1F51**	1IXM	1SRR	35%	35%	30%	30%
**1KAC**	1NOB	1F5W	30%	30%	30%	30%
**1HE1**	1HE9	1MH1	35%	35%	30%	30%
**1R0R**	2GRK	1SCN	30%	30%	30%	30%
**1BUH**	1HCL	1DKS	35%	35%	35%	35%
**1B6C**	1D6O	1IAS	35%	35%	30%	30%
**1GPW**	1THF	1K9V	35%	35%	35%	35%
**1K74**	1MZN	1ZGY	30%	30%	30%	30%
**2HQS**	1CRZ	1OAP	30%	30%	30%	30%
**1DFJ**	9RSA	2BNH	35%	35%	35%	35%
**1TMQ**	1JAE	1B1U	35%	35%	35%	35%
**1KXP**	1IJJ	1KW2	35%	35%	30%	30%
**2HRK**	1HRA	2HQT	30%	30%	30%	30%
**1R6Q**	1R6C	2W9R	40%	40%	30%	30%
**1KKL**	1JB1	2HPR	35%	35%	35%	35%
**1ACB**	1egl	2cga	40%	40%	30%	30%
**1GRN**	1A4R	1RGP	40%	40%	30%	30%
**1IB1**	1KUY	1QJB	35%	35%	30%	30%
**2Z0E**	2D1I	1V49	40%	40%	30%	30%
**2JIW**	1AKL	2RN4	40%	40%	30%	30%
**2J7P**	1NG1	2IYL	40%	40%	35%	35%
**1GP2**	1GIA	1TBG	40%	40%	30%	30%
**2O3B**	1ZM8	1J57	30%	30%	30%	30%
**1F6M**	1CL0	2TIR	35%	35%	35%	35%
**1IRA**	1ILR	1G0Y	40%	40%	35%	35%
**1PXV**	1X9Y	1NYC	35%	35%	30%	30%
**2C0L**	1FCH	1C44	40%	40%	35%	35%

Table S4: **Cutoff selection for BLUEPRINT analysis**. Each line reports the cutoff value on the total number of coupled (red) or uncoupled (blue) residues accepted after sorting as part of the final results for each protein/complex, in all testing conditions: monomers/uncoupled, complex/uncoupled, monomers/coupled, complex/coupled. A cutoff value higher than the average (35%) may be required in cases featuring a conformational displacement between isolate and bound forms, such as “medium” cases (grey shading) and “difficult” ones (dark grey shading)

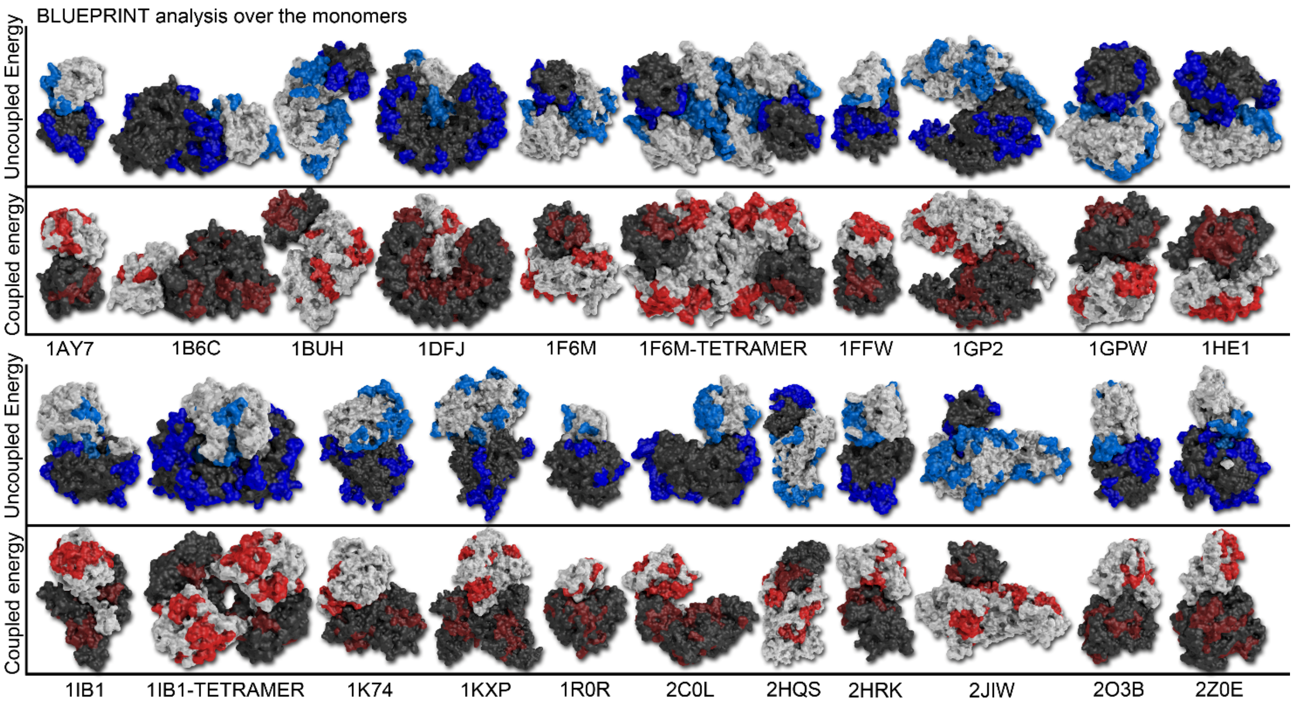

Figure S1: **Visual representation of BLUEPRINT results from the analysis of 38 monomers, mapped on the 3D structure of the full dimers and tetramers**. This figure depicts a visual representation of the results of BLUEPRINT analysis for the remaining proteins of the dataset, which could not be shown in Figure 3 or Figure 4. For every pair of protein, the PDB ID of the complex is located on the bottom line, and the series of molecular renders is subdivided between energy uncoupling (upper row, blue stripes) and coupling analyses (lower row, red stripes). In order to display the results clearly, the view of the complexes is usually rotated going from uncoupled to coupled analysis, so upper and lower complexes may appear different. The two proteins composing each dimer are identified by a different color (white and dark gray). The color code is maintained from Figure 3: light blue/red regions belong the white monomer, while deep blue/red regions belong to the dark one.

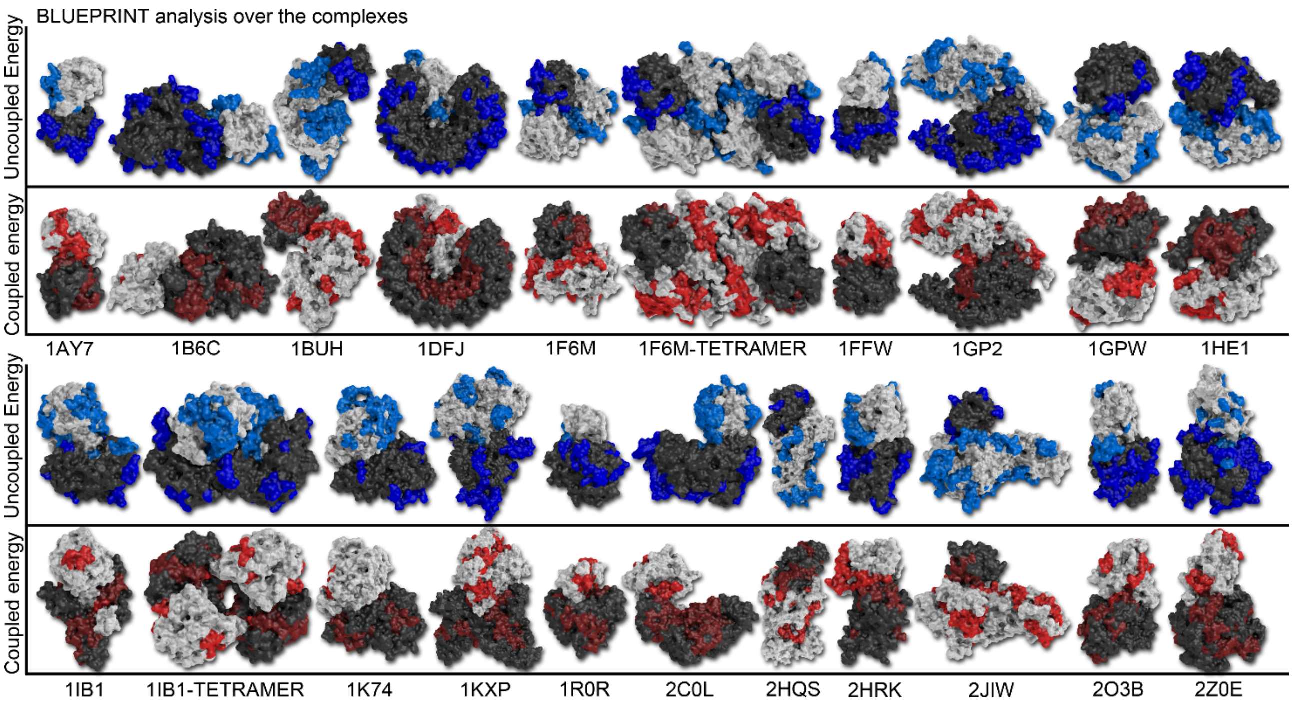

Figure S2: **Visual representation of BLUEPRINT results from the analysis of 19 dimers**. This figure shows the results of BLUEPRINT analysis performed over the full dimers for those proteins of the dataset which could not be integrated in Figures 3 and 4. Two cases (1F6M and 1IB1) display also a tetrameric version, showing the result of complex analysis (dimers) mapped on the structure of their full biological assembly.
